# Expansion of LINEs and species-specific DNA repeats drives genome expansion in Asian Gypsy Moths

**DOI:** 10.1038/s41598-019-52840-z

**Published:** 2019-11-11

**Authors:** Francois Olivier Hebert, Luca Freschi, Gwylim Blackburn, Catherine Béliveau, Ken Dewar, Brian Boyle, Dawn E. Gundersen-Rindal, Michael E. Sparks, Michel Cusson, Richard C. Hamelin, Roger C. Levesque

**Affiliations:** 1 Institut de Biologie Intégrative et des Systèmes (IBIS), Université Laval, Ville de Québec, Canada; 2 Laurentian Forestry Centre, Canadian Forest Service, Natural Resources Canada, Quebec City, Quebec Canada; 3 Department of human genetics, McGill University, Montreal, Quebec Canada; 4 United States Department of Agriculture - ARS Invasive Insect Biocontrol and Behavior Laboratory, Beltsville, Maryland USA; 5 Department of Forest and Conservation Sciences, Faculty of Forestry, University of British Columbia, Vancouver, British Columbia Canada

**Keywords:** Computational biology and bioinformatics, Genome

## Abstract

Two subspecies of Asian gypsy moth (AGM), *Lymantria dispar asiatica* and *L. dispar japonica*, pose a serious alien invasive threat to North American forests. Despite decades of research on the ecology and biology of this pest, limited AGM-specific genomic resources are currently available. Here, we report on the genome sequences and functional content of these AGM subspecies. The genomes of *L.d. asiatica* and *L.d. japonica* are the largest lepidopteran genomes sequenced to date, totaling 921 and 999 megabases, respectively. Large genome size in these subspecies is driven by the accumulation of specific classes of repeats. Genome-wide metabolic pathway reconstructions suggest strong genomic signatures of energy-related pathways in both subspecies, dominated by metabolic functions related to thermogenesis. The genome sequences reported here will provide tools for probing the molecular mechanisms underlying phenotypic traits that are thought to enhance AGM invasiveness.

## Introduction

North American forests face unprecedented threats from multiple Forest Invasive Alien Species (FIAS) that can potentially cause large-scale disturbances and major biological, social, and economic impacts. One of the most threatening FIAS currently identified in Canada and the United States is the Asian gypsy moth (AGM). The term AGM refers to a group of closely related species of *Lymantria* moths (order Lepidoptera), including *L. dispar asiatica*, *L. dispar japonica*, *L. umbrosa*, *L. albescens*, and *L. postalba*^1–3^. Although the European gypsy moth (EGM; *Lymantria dispar dispar*) became established in North America more than 100 years ago, AGM are currently considered “non-established”. However, AGM represent a constant threat as they have often been intercepted at North American ports. AGM also exhibit several ecological characteristics that may increase their invasive capacity compared to EGM. First, they have a broad host range (>500 botanical species), including more coniferous species than EGM^4^. Second, they are attracted to light, which makes them more inclined to oviposit on cargos and ships that transport goods around the world^5^. Third, eggs of AGM subspecies require shorter chilling time before hatching^6,7^. As a result, increasing temperatures due to climate change may help spread AGM subspecies into new geographic regions. Fourth, unlike EGM, AGM females are capable of direct and sustained flight and so can disperse on a larger spatial scale^5,8^. Overall, these features likely enhance AGM invasion capacity. Notably, AGM and EGM can also successfully interbreed, producing flight-capable and fertile hybrid progeny^9^. Monitoring the large-scale dispersal patterns of these insects around the world could help reduce their impact on forest ecosystems.

The ability to predict gypsy moth female invasiveness is of critical importance in developing and implementing adequate and efficient management programs. Yet, despite considerable research efforts invested in understanding behavioral and ecological aspects of *Lymantria* moths, the molecular mechanisms responsible for the expression of key invasive traits in this species complex remain largely uncharacterized. Only partial transcriptome sequences are available for EGM^10^ and *L.d. asiatica*^11,12^. Although a draft genome sequence was recently published for EGM^13^, no whole genome sequences currently exist for any AGM subspecies.

Here we present gypsy moth reference genomes for *L.d. asiatica* and *L.d. japonica*, and we describe their gene content and organization. We show that these AGM subspecies possess the largest genomes sequenced to date among lepidopterans, a characteristic that can be explained by the accumulation of numerous transposable elements. The reference genomes reported here provide valuable genomic information that can be used for gene mapping, comparative analyses, development of genome engineering tools, as well as to better understand key aspects related to the biological characteristics that make AGM one of the greatest threats to North American forests.

## Results and Discussion

### Largest sequenced lepidopteran genome

**Figure 1:**
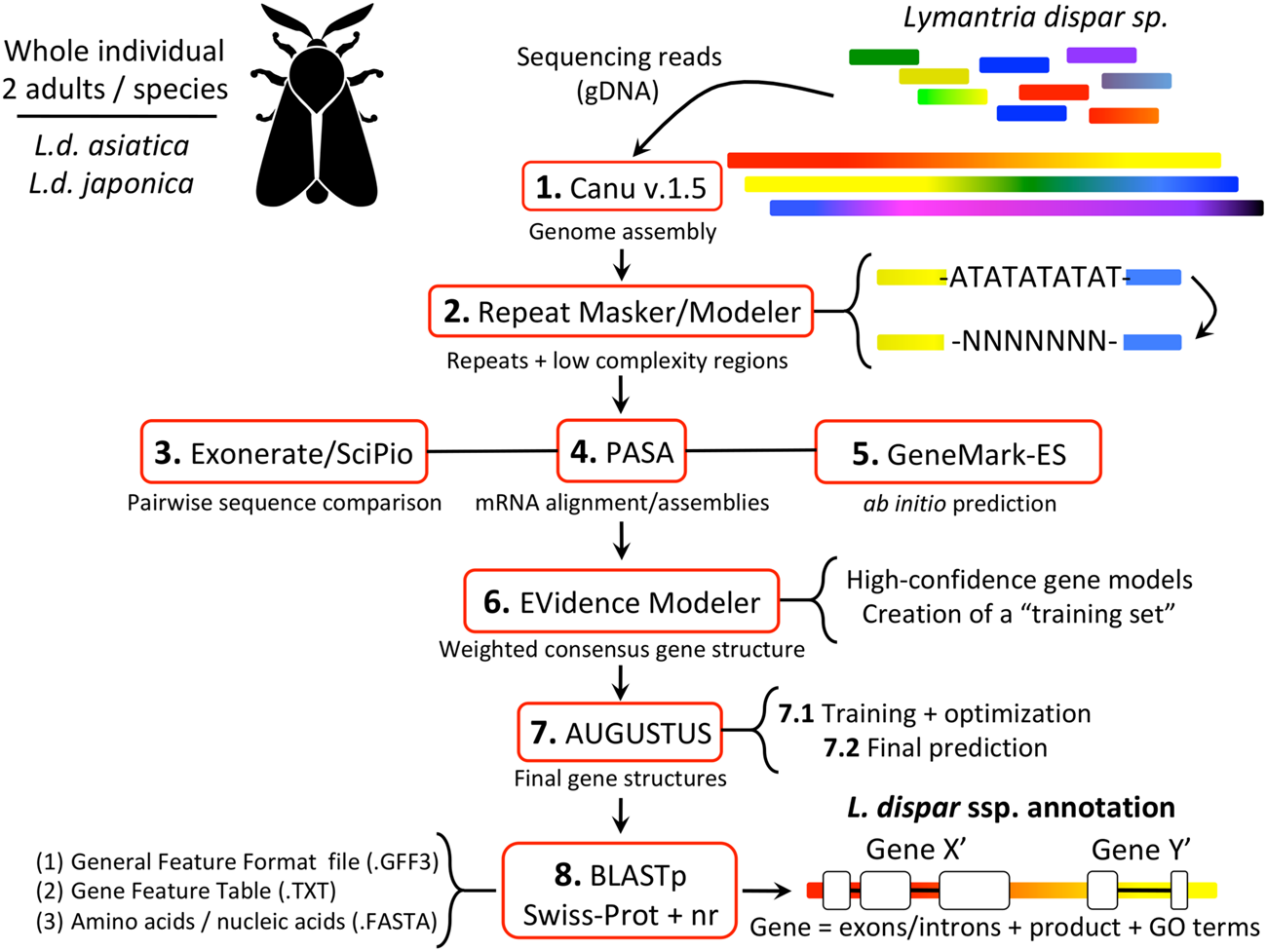
Genome annotation pipeline specifically optimized and trained to identify gene structures in Lepidopteran genomes.

Through our in-house assembly and annotation pipeline (Fig. 1, see Supplementary Information Section S2), we sequenced the genome of *L.d. asiatica* using DNA extracted from two adult males, with 70 PacBio RSII SMRT cells and the P6-C4 chemistry. Our pipeline was trained and optimized to specifically annotate moth genomes (Fig. 1, step 7.1 – Augustus training). This yielded 8.5 million raw sequencing reads for a total of 35.6 Gb sequenced, with uncorrected genome coverage of 36 × (CANU assembly report). Similarly, DNA from two adult *L.d. japonica* males was used to generate 44 PacBio RSII SMRT cells (also with P6-C4 chemistry), yielding 5.4 million raw sequencing reads, for a total of 30.6 Gb sequenced, with uncorrected genome coverage of 31 × (CANU assembly report). Post-processing steps returned total genome sizes of 921 Mb (final coverage = 47X) and 999 Mb (final coverage = 37X), with N50s of 212 Kb and 137 Kb, for *L.d. asiatica* and *L.d. japonica*, respectively (Table 1, see Supplementary Information Sections S2 and S3). Previous work on genome sizes in the Lepidoptera established that the total genome size of *L. dispar dispar* (EGM) was ~1,007 Mb^14^. Additional flow cytometry-based EGM genome size determinations generated comparable results (male genome size of 993.3 ± 6.2 Mb and female genome size of 983.2 ± 6.7 Mb; work conducted in the laboratory of Dr. Spencer Johnston, Texas A&M University, United States). This result provides indirect corroboration for our genome size estimates in AGM and suggests a high level of completeness for the AGM assemblies. A k-mer distribution analysis performed on both genomes revealed that eight percent (*L.d. asiatica*) and 10 percent (*L.d. japonica*) of the k-mers that we generated were present in more than one copy, which suggests a low level of potential allelic contigs in our assemblies (see Supplementary Information Section S3 and Supplementary Fig. S1). The total genome sizes and low level of allelic duplicates indicate that the genomes generated in this study can be considered as subspecies-level references. This will ensure that each reference genome contains the least amount of errors. The 78 Mb difference between *L.d. asiatica* and *L.d. japonica* genome sizes may be explained in part by significant differences in raw sequencing read lengths in each of the two independent sequencing and assembly procedures. Corrected read length distributions showed that *L.d. japonica* had a read N50 of 11.9 Kb, while *L.d. asiatica* had a read N50 of 9.4 Kb (Supplementary Fig. S2), which could explain why we obtained a larger genome assembly in the case of *L.d. japonica*. In addition, more Arthropoda BUSCO (Benchmarking Universal Single Copy Orthologs^15^) genes were captured in the *L.d. japonica* assembly (98.2% total BUSCO coverage) compared to that of *L.d. asiatica* (96.5% total BUSCO coverage), consistent with the observation that the *L.d. japonica* genome is larger and closer to the expected genome size than the *L.d. asiatica* genome (Supplementary Information Section S2). These results expand the range of reported genome assembly sizes of related lepidopteran species such as the urticating pine defoliator (*Thaumetopoea pityocampa*, 537 Mb^16^), the silk worm (*Bombyx mori*, 481 Mb^17^), the greater wax moth (*Galleria mellonella*, 578 Mb^18^), the Japanese oak silk moth (*Antheraea yamamai*, 656 Mb^19^), the common wood-white moth (*Leptidae sinapis*, 643 Mb^20^), and the red-banded hairstreak (*Calycopis cecrops*, 729 Mb, according to http://lepbase.org ^21^). The largest lepidopteran genome sequenced before this study was the genome of the closely related EGM (*Lymantria dispar dispar*, 865Mb^13^), making the AGM genomes obtained here the largest lepidopteran genomes sequenced to date.

**Table 1:** Composition of the two AGM *Lymantria dispar* spp. genomes sequenced in this study in comparison to the sequenced genomes of EGM *Lymantria dispar dispar* and the well characterized sister species *Bombyx mori*.

Feature	Bmo*	Ldd^▯^	Lda	Ldj
Total size (Mb)	481	865	921	999
Contigs	88,637	194,709	8,190	11,303
Scaffolds	43,463	134,446	n.a.	n.a.
N50 (Kb)	4,008	5,068	212	137
N90 (Kb)	0.262	0.245	73	82
Repeat (%)	43.6	36	59.9	59.6
GC content (%)	37.7	35.2	38.7	38.5
Protein-coding genes	15,488	13,331	19,588	23,292
Exon (%)	4.7	1.8	2.7	3.0
Intron (%)	16.1	17	27.6	28.4
BUSCO coverage (%)	95.5	89.2	96.5	98.2

Abbreviations: Bmo, Bombyx mori; Ldd, Lymantria dispar dispar; Lda, Lymantria dispar asiatica; Ldj, Lymantria dispar japonica.

^*^Reference genome ASM15162v1, accession number GCA_000151625.1 (http://ensembl.lepbase.org/), described in^17^.

^▯^Numbers for this genome were taken from Zhang *et al*.^13^. Percentages of exon and introns for Bmo and Ldd were taken from this reference, while the values for Lda and Ldj were calculated based on the GFF3 files produced in this study.

BUSCO values were obtained form BUSCO v.3.0^15^. Reported values correspond to total BUSCO genes retrieved, i.e. complete (single + duplicated) and fragmented.

**Figure 2:**
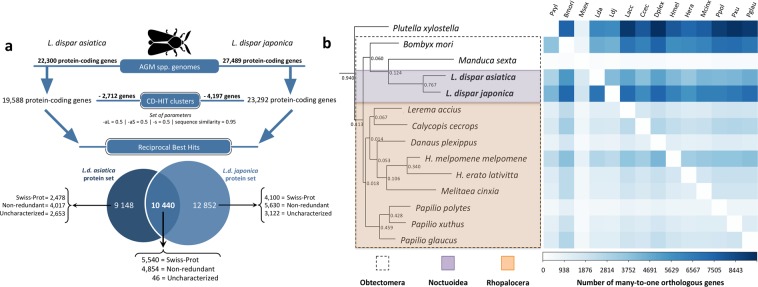
Genome assembly post-processing steps. (**a**) Gene models were refined by (i) reducing redundancy using the program CD-HIT and (ii) assessing protein sequence similarities between *L.d. asiatica* and *L.d. japonica* using a reciprocal best hit approach with BLASTp. (**b**) Rooted species tree inferred from gene trees generated by orthoFinder for all amino acid orthogroups shared among 14 Lepidopteran taxa (including AGM) encompassing the Noctuoidea (orange) and Rhopalocera (violet) superfamilies of the Obtectomera clade (dashed box). The heatmap on the right represents pairwise comparisons between all species included in the analysis, showing the number of many-to-one (N:1) orthologous genes between each species pair.

Gene sets exhibited numbers of protein-coding genes within the range found in other Lepidopteran genomes (between 10,117 and 29,902 genes, see Supplementary Table S3 for details). Genome assembly post-processing steps, focused on the reduction of redundancy through sequence similarity, allowed us to identify the structure of 19,588 and 23,292 genes in *L.d. asiatica* and *L.d. japonica* respectively (Fig. 2a, Supplementary Information Section S3). Similar proportions of KEGG KO categories identified between the two genomes suggest that they are very similar in terms of overall functional content (Supplementary Fig. S3). While the difference in the numbers of genes we identified in the two subspecies was unexpectedly high, the greater value obtained for *L.d. japonica* may be attributable (at least in part) to technical artifacts, resulting in a higher degree of artificial duplication in the assembly of its genome, as suggested by the higher BUSCO score obtained for the “complete & duplicated” hits (Supplementary Fig. S4). Nonetheless, the possibility that *L.d. japonica*’s genome does feature a greater number of genes than that of *L.d. asiatica* should not be overlooked. A complementary reciprocal best hit (RBH) analysis performed during post-processing steps further revealed that 10,440 genes possess nearly identical protein sequences between *L.d. asiatica* and *L.d. japonica*, of which 99.6% were annotated with a putative gene product (Fig. 2a, Supplementary Table S4). This additional layer of information adds to the existing gene product, gene ontology and KEGG functional pathway assignment. It can be used as an extra measure of confidence that a given gene is not a false positive because it has been characterized in multiple complementary analyses. An additional orthologous analysis comparing whole genome sequences of 12 lepidopteran species with the AGM genomes sequenced here corroborated previous phylogenetic relationships among these taxa^22–24^ (Fig. 2b, Supplementary information S3). The analysis also confirmed the highly repeated nature of AGM genomes, showing numerous many-to-one orthologous relationships with other lepidopteran taxa, which explains some of the low bootstrap values obtained in the final rooted species tree generated by orthoFinder (Fig. 2b). These results suggest that despite the presence of numerous repetitive sequences, gene models generated for each of the two AGM subspecies remain of high quality. Complimentary gapped BLASTn analyses of the published EGM transcriptome^10^ against our AGM genomes revealed that 98% and 99% of the transcripts align onto the genome of *L.d. asiatica* and *L.d. japonica*, respectively (with an average 99% identities for both genomes). Overall, the present results indicate that the genomes generated in this study can be used as reliable draft reference assemblies. Future genome versions will incorporate data from complementary sequencing platforms to increase N50s and polish these reference sequences.

### High prevalence of transposable elements in *L. dispar* spp. genomes

**Figure 3:**
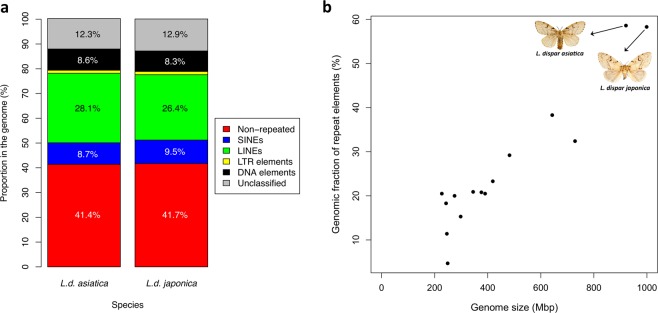
High content of repeated sequences in AGM. (**a**) Proportion of specific repeated element categories in *L.d. asiatica* (black) and *L.d. japonica* (white) genome assemblies. (**b**) Total percentage of repeat elements in the genome is positively correlated with genome size across Lepidopteran species. Data on genome sizes and repeat elements from 13 Lepidopteran species (*Bombyx mori, Calycopis cecrops, Danaus plexippus, Heliconius melpomene, Leptidea sinapis, Lerema accius, Manduca sexta, Melitaea cinxia, Papilio glaucus, Papilio polytes, Papilio xuthus, Papilio sennae, Papilio rapae*) were taken from Talla *et al*.^20^ (see Supplementary Table S3). *L.d. asiatica* photograph taken by Alexander Schintlmeister and *L.d. japonica* photograph taken by Ken Walker for the Museum Victoria, PaDIL (CC BY 3.0 au).

The AGM genomes assembled contained large proportions of repetitive DNA spread across all contigs, most of which are transposable elements (TEs) described in other animal species. Nearly 60% of the genomes of *L.d asiatica* and *L.d. japonica* consist of TEs and repeated sequences. Long Interspersed Nuclear Elements (LINEs) are the most abundant TEs identified in both genomes, accounting for 28% and 26% of all genomic DNA (gDNA) in *L.d. asiatica* and *L.d. japonica*, respectively (Fig. 3a). Interestingly, the second most abundant group of repeated elements (12% and 13% of all gDNA in *L.d. asiatica* and *L.d. japonica*, respectively) were novel, “unclassified” elements that were identified through the creation of a *de novo* repeat library (Supplementary Information Section S2).

Short Interspersed Nuclear Elements (SINEs) are the third most abundant TEs, comprising10% and 9% of *L.d. asiatica* and *L.d. japonica* genomes, respectively. Both AGM genomes exhibit considerable difference in total proportion of TEs and occurrence of certain TE classes when compared to most other Lepidopteran taxa. The largest proportion of TEs observed in a lepidopteran genome before this study was in *Leptidea sinapis* (38.3%), while other taxa exhibit total TE proportions between 4.7% and 29.2%^20^. The proportion of LINEs in the two AGM assemblies are particularly high compared to other lepidopteran species, for which estimates vary from 0.7% (common Mormon, *Papilio polytes*) to 5.4% (white-wood butterfly, *Leptidea sinapis*)^20^. The results presented here for *L.d. asiatica* and *L.d. japonica* support and extend a strong correlation (Pearson’s; n = 15, R = 0.95, r^2^ = 0.908, P-value = 4.25 × 10^−8^) between genome size and repeat content in lepidopteran genomes (Fig. 3b, Supplementary Table S2). This relationship has also been reported for insects^25^, fishes^26,27^, flowering plants^28^, birds and mammals^29^, and trees^30,31^.

The pronounced expansion of LINEs and unclassified elements in AGM genomes supports the idea that TE activity plays a strong role in genome size evolution in the Lepidoptera, and potentially in eukaryotes in general. This could have significant functional implications in terms of gene regulatory networks, as TE-derived sequences may be co-opted for cis-regulatory elements in the genome^32–35^. Recent research on both prokaryotic and eukaryotic invasive species suggests that interactions between environmental stresses and activity of transposable elements could fuel rapid adaptation to new environments and promote invasiveness through genomic structural changes or other innovations^36–39^. Characterizing TEs diversity and identifying their precise genomic locations in AGM could also help develop a foundation for exploring gypsy moth gene regulation.

### Reconstruction of metabolic pathways for different insect species

**Figure 4:**
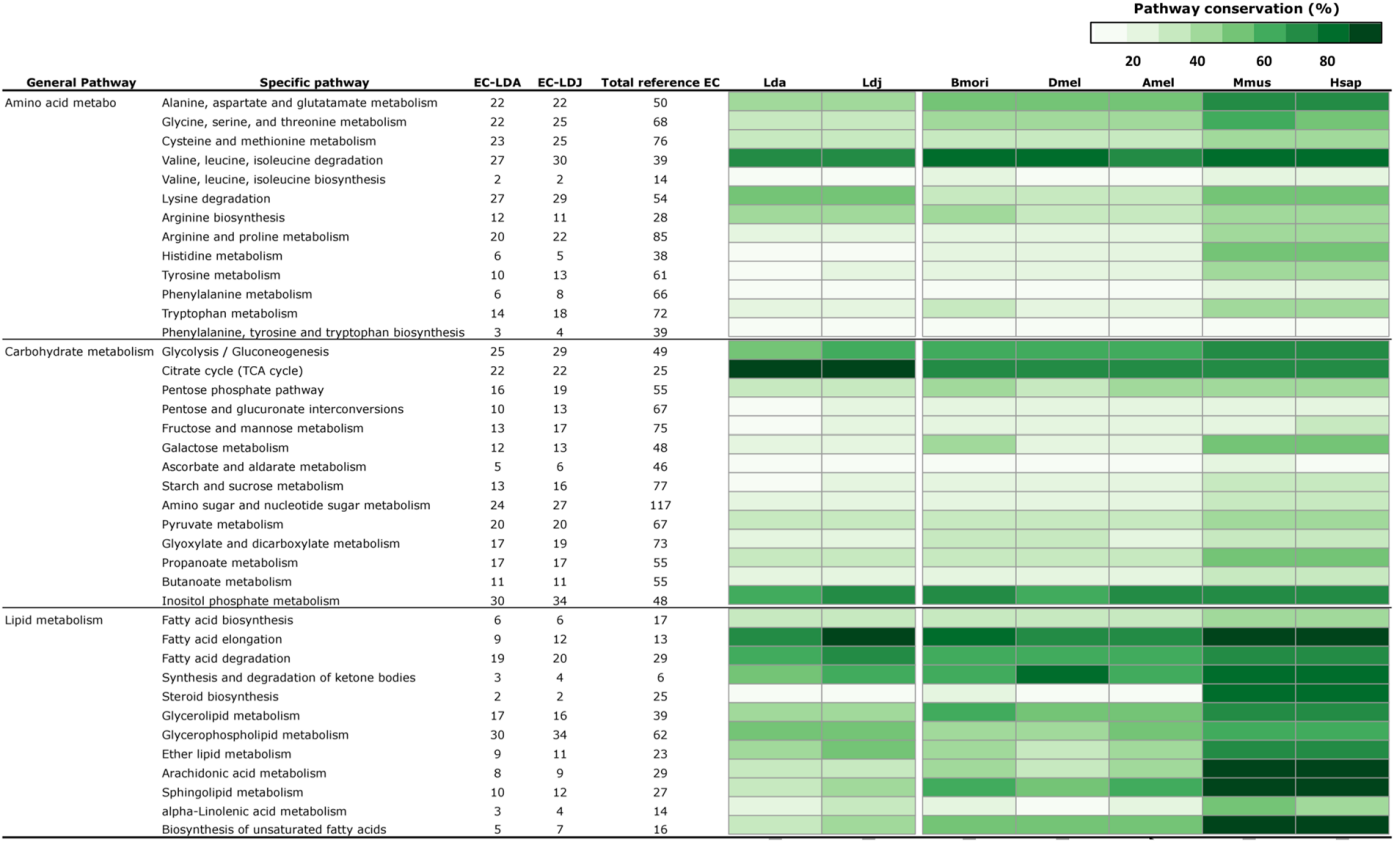
Conservation of genome-wide KEGG metabolic pathways. Characterization and comparisons of metabolic pathways across three different insect orders (*Bombyx mori* – Lepidoptera, *Apis mellifera* – Hymenoptera, *Drosophila melanogaster* – Diptera) and two well-characterized mammalian species (*Mus musculus* and *Homo sapiens*) in comparison with *L.d. asiatica* and *L.d. japonica* (Lepidoptera). Rows represent specific metabolic pathways grouped in three general categories, i.e. amino acid metabolism, carbohydrate metabolism, and lipid metabolism, as defined by the Kyoto Encyclopedia of Genes and Genomes (KEGG). Colors represent the species-specific pathway conservation level, defined as the percentage of enzymes identified in the genome as compared to the KEGG reference pathway. Amel = *Apis mellifera*, Bmori = *Bombyx mori*, Dmel = *Drosophila melanogaster*, Lda = *Lymantria dispar asiatica*, Ldj = *Lymantria dispar japonica*, Hsap = *Homo sapiens*.

Metabolic pathway reconstruction analysis showed a relatively high conservation level in most pathways across various taxa, with small differences in several pathways in AGM (Fig. 4). A recent large-scale metabolic investigation in AGM, based on transcriptomics, revealed a significantly greater number of unique transcripts associated with chitin degradation than chitin biosynthesis, two crucial processes taking place during growth and development^12^. We confirmed this trend, identifying 32 genes involved in chitin catabolism (mostly chitinases) and 16 genes involved in chitin biosynthetic pathways (Supplementary Tables S5 and S6). Considering that chitin is the major biochemical component of insect cuticle that must be produced and degraded rapidly during molting phases at the larval stage (the developmental stage at which intense forest defoliation occurs during AGM outbreaks), characterizing genes involved in chitin-related metabolism offers promising molecular targets from which new species-specific biocontrol strategies could be developed^40^ (e.g., blocking chitin metabolism to prevent growth and development). Another aspect of chitin-related genes that may have major implications for AGM invasiveness is cold tolerance. Transcriptomics results obtained from eight species of stick insects^41,42^ and in the seabuckthorn carpenter moth (*Eogystia hippophaecolus*, see^43^) suggested a parallel pathway to cold tolerance adaptation based on cuticle-related genes. The three gene products that systematically responded with high levels of expression at low temperature^41^ (prolyl 4-hydroxylase subunit alpha-1, staphylococcal nuclease domaincontaining protein 1 and a cuticular protein gene), along with 90 and 103 cuticle-related gene products, have been fully annotated in the present *L.d. asiatica* and *L.d. japonica* genome assemblies, respectively (Supplementary Tables S5 and S6).

Specific pathways related to chitin metabolism are part of a more global “carbohydrate metabolism” pathway, which also features the most conserved pathway identified in AGM subspecies: the citrate cycle (TCA cycle or Krebs cycle, Fig. 4). This citrate cycle performs the last steps of the oxidation of carbohydrates and fatty acids. In total, 22 enzymes belonging to this pathway, out of the 25 enzymes that compose the full KEGG reference pathway, were identified in the genomes of *L.d. asiatica* and *L.d. japonica*. In comparison to the other taxa included in the analysis, this result indicates that AGM genomes contain diversified enzymatic tools associated with several biochemical pathways regulating energy production.

Thermogenesis is another energy-related metabolic pathway that we explored and that could play a significant role in the determination of flight capacity and cold tolerance in AGM (KEGG pathway number ko04714). This pathway came second in number of successful hits on the KEGG database. In total, 102 and 114 unique KEGG numbers were identified in the genomes of *L.d. asiatica* and *L.d. japonica*, respectively. These were associated with 19 (*L.d. asiatica)* and 23 (*L.d. japonica*) unique EC enzymes involved in thermogenesis (Supplementary Table S7). This result suggests that in AGM, the importance of energy metabolism may also be closely linked with its flight capability, which requires high fluxes of energy executed at high core metabolic temperatures^44,45^. Moths are recognized for their high capacity for evasiveness during flight, having the ability to quickly initiate flight, attain varying speeds and change direction rapidly and erratically^46^. Moths are also heterothermic, which means they cannot perform these energy-consuming activities efficiently at low ambient temperatures^47^. As a result, AGM likely need to control their body temperature with high precision through various regulatory mechanisms in order to fly^48^. In insects, thermogenesis is activated during multiple biological activities, such as flying, running, singing, pre-flight warm-up, and social activities^49^. In this context, thermogenesis and locomotive functions are closely connected. Disentangling these issues will require specific hypothesis-driven functional experiments, and the genome assemblies reported here provide a key resource to meet that challenge. AGM genomes exhibit a broad metabolic competence by encoding a wide variety of enzymes required to perform flight, feed on multiple different hosts, and tolerate cold temperatures, traits strongly implicated in the invasive nature of these insects.

### Genome-wide characterization of developmental genes

One core question pertaining to the ecological differences between AGM subspecies is how and why traits associated with flight capability vary in different *Lymantria dispar* ssp. populations. Data on wing morphometrics from eight geographic populations of *Lymantria dispar* spp., including EGM and AGM subspecies, has shown that females from populations with strong directed flight capabilities have significantly lower wing load (i.e. body mass divided by total wing area) than females from flightless populations^50^. These phenotypic differences, along with differences in population-specific flight muscle capacities, can predict flight capabilities in individuals from unknown source populations. Since flight-related muscle function is strongly correlated with the flightless phenotype, we assessed the possible presence of a loss-of-function mutations within the coding sequence of flightin (*fln*), a gene required for proper direct flight muscles assembly in *Drosophila melanogaster*^51^. In other species, coding sequence mutations have been implicated in sexually dimorphic trait expression when regulated by modifiers or dosage compensation^52,53^. However, we found no evidence for *fln* coding sequence evolution underlying flight capability differences between EGM and AGM (see Supplemental Information, Section S5 for further details).

We also characterized the presence and structure of homeotic genes in the AGM genomes (summarized in Supplementary Fig. S5), in order to test the possibility that differences in female flight capability are the result of different developmental trajectories between flight capable and flight incapable individuals. Homeotic genes encode crucial transcription factors involved in body patterning and developmental trajectories^54^. These genes could harbor intrinsic sequence differences or gene copy numbers among AGM subspecies that could be involved in different developmental trajectories, resulting in different wing loads or varying muscle strength capacities. We identified a collection of 87 (*L.d. asiatica*) and 111 (*L.d. japonica*) homeotic genes, spanning a total of 60 unique homeobox gene families and distributed across ten different gene classes. No major structural rearrangements (e.g. cluster organization, expansion/retraction of certain families) were detected between homeotic genes identified in AGM, as compared to other Lepidopteran species (see Supplementary Information Section S4 for further details). Our description of AGM hox and other homeotic genes however supplies key resources for exploring other potential developmental causes to differential flight capacity (e.g. population-specific SNPs in key developmental genes), and appendage formation in gypsy moths and the Lepidoptera in general.

## Conclusion

Although AGM moths are not currently established in North America, they represent a constant threat to global forest ecosystem stability due to their strong invasive capacity. The novel reference genomes presented in this study provide valuable genomic information that can be used to better understand the biological characteristics that make AGM one of the top threatening forest pests in North America. This study also shows that it is possible to assemble a large and complex genome with a single long read dataset, in this case PacBio. It shows that smaller research teams with limited resources can build reliable reference genomes that match most of the high-quality genomic references obtained with more elaborate datasets. Moths and butterflies show variable genome sizes and complexities. Our genome assembly pipeline, specifically developed to recognize lepidopteran genes, revealed that AGM possess the largest genomes sequenced to date among Lepidoptera, a characteristic that can be explained by the accumulation of species-specific transposable elements. Large-scale genomic data generated in this study will help in the identification of the genetic basis of key traits defining the invasive capacity of AGM, such as host diet breadth, cold tolerance, and female flight capacity. Population-scale investigations based on the reference assemblies generated here will also enable the development of highly specific molecular diagnostic tools to create fine-tuned monitoring and managing strategies for future AGM outbreaks.

## Methods

### Sequencing, genome assembly and annotation

Genome sequencing was carried out using the Single Molecule, Real-Time technology (SMRT) developed by Pacific Biosciences (PacBio). We developed an 8-step custom-made genome assembly and annotation pipeline available on Github (https://github.com/fohebert/GenomeAnnotation, see Supplementary Information Section S2 for further details). Complete raw sequence data obtained from four different specimens (Supplementary Information Section S2) was registered under the NCBI BioProject IDs PRJNA479680 (*L.d. asiatica*) and PRJNA479831 (*L.d. japonica*), associated with NCBI Sequence Read Archive (SRA) accession numbers SAMN09601828 (*L.d. asiatica*) and SAMN09601829 (*L.d. japonica*). Steps in the pipeline include (1) the assembly of raw sequencing reads into longer contig sequences using Canu v.1.5 (genomeSize = 1.0 g, maxMemory = 800, maxThreads = 60, all other parameters kept to default mode^55^). (2) Identification and masking of repeated DNA. A *Lymantria*-specific repetitive DNA database was first created using RepeatModeler v.1.0.8^56^ (‘-engine ncbi’, all other parameters set at default values) and was then used in combination with the curated Repbase library of repeats^57^ to screen the AGM genomes for specific classes of repeats using RepeatMasker v.4.0.6^58^. (3) Identification of protein-coding genes in AGM genomic sequences through pairwise sequence comparison. This step was conducted through two complementary approaches implemented in the programs Exonerate v.2.4.0^59^ and SciPio v.1.4.1^60^. (4) *Lymantria*-specific mRNA transcript alignments onto each of the two AGM genomes using PASA v.2.1.0^61^. (5) Genome-wide *ab initio* identification of gene sequences using the program GeneMark-ES v.1.0^62^. (6) Computation of weighted consensus gene structure annotations based on the evidence gathered through steps three to five, using EVidenceModeler (EVM) v.1.1.1^63^. (7) Genome-wide identification of protein-coding genes by training AUGUSTUS v.3.2.2^64^ on a high-quality gene set identified through EVM in the previous step, and then running the trained version of the program on the complete AGM genomes. (8) Assignment of gene products and Gene Ontology (GO) terms to AGM protein-coding genes using BLASTp searches in NCBI’s UniprotKB/Swiss-Prot and non-redundant protein databases. Post-processing steps were also performed to polish final contig sequences using PacBio’s GenomicConsensus package (https://github.com/PacificBiosciences/GenomicConsensus) and eliminate redundancy in gene models (see Supplementary Information Section S3 for further details). We ultimately clustered similar amino acid sequences into consensus gene models using CD-HIT^65^ and performed whole-genome sequence comparisons with 12 other Lepidopteran species to identify orthology relationships, using orthoFinder^66^ (Supplementary Information Section S3).

### Metabolic pathway reconstruction

We assigned KO (KEGG Orthology) numbers to the amino acid sequences obtained through our assembly pipeline using the BlastKOALA annotation server^67^, which allowed the reconstruction of various molecular networks such as carbohydrate, lipid and amino acid enzymatic pathways. To perform this analysis, we retrieved the Enzyme Nomenclature (EC) numbers based on the KO numbers (ECs are attributes of KEGG Orthology numbers) in the raw BlastKOALA output, and estimated the percentage of conservation for each specific pathway, defined as the percentage of enzymes identified in a species-specific pathway as compared to the KEGG reference pathway. We compared the results to five other species: *Bombyx mori* (silk worm); *Drosophila melanogaster* (fruitfly); *Apis mellifera* (European honey bee); *Mus musculus* (mouse), and; *Homo sapiens* (human).

### Identification and phylogeny of homeotic genes

Complete sets of homeotic genes from 10 different animal species were downloaded from the HomeoDB^2^ website^68^: human (*Homo sapiens*); mouse (*Mus musculus*); chicken (*Gallus gallus*); frog (*Xenopus* [*Silurana*] *tropicalis*); zebrafish (*Danio rerio*); amphioxus (*Branchiostoma floridae*); fruitfly (*Drosophila melanogaster*); red flour beetle (*Tribolium castaneum*); western honeybee (*Apis mellifera*), and; nematode (*Caenorhabditis elegans*). We used these gene sequences to build a local database to which all of the amino acid sequences predicted from the genomes of *L.d. asiatica* and *L.d. japonica* were compared, using the sequence similarity algorithm implemented in NCBI’s BLASTp program v.2.6.0^69^. BLASTp results exhibiting an e-value < 1e-15 and an overall identity value >40% were considered significant and were kept for downstream phylogenetic analysis. All significant BLASTp results were further manually validated using the Simple Modular Architecture Research Tool (SMART)^70,71^, in combination with a visual assessment of the BLASTp result to confirm that the candidate homeotic genes truly contain at least one homeodomain sequence. Pseudogenes were annotated but discarded from downstream analyses. Manually validated homeodomain sequences resulting from the sequence homology analysis were aligned using CLUSTAL-OMEGA v.1.2.4^72^ with mBed-like clustering guide-tree and iteration, a maximum of five combined iterations, five guide tree iterations, and five Hidden Markov Model (HMM) iterations (all other parameters set at default values). The resulting alignment was used to construct a phylogenetic tree with RAxML v.8.2.0^73^, following a gamma model rate of heterogeneity, combined with a WAG substitution matrix and a maximum likelihood search of 100 bootstraps. Final trees were generated and edited using FigTree v.1.4.3 (http://tree.bio.ed.ac.uk/software/figtree/).

## Supplementary information

Supplementary information document

Supplementary table S1

Supplementary table S2

Supplementary table S3

Supplementary table S4

Supplementary table S5

Supplementary table S6

Supplementary table S7

Supplementary table S8

Supplementary table S9

## Data Availability

The raw sequencing files used to generate the genome assemblies analyzed during the current study were registered as NCBI BioProject IDs PRJNA479680 (*L.d. asiatica*) and PRJNA479831 (*L.d. japonica*), associated with NCBI Sequence Read Archive (SRA) accession numbers SAMN09601828 (*L.d. asiatica*) and SAMN09601829 (*L.d. japonica*). The genome assembly pipeline used to analyze the raw datasets, with corresponding Perl, Python and bash utility scripts, as well as final FASTA/GFF3 genome files, are available on Github: https://github.com/fohebert/GenomeAnnotation, as well as on the Open Science Framework public repository: 10.17605/OSF.IO/UNZ2V. All other data generated/analyzed during this study are otherwise included in this published article and its Supplementary Files.
