# Supplementary material for: Expansion of LINEs and species-specific DNA repeats drives genome expansion in Asian Gypsy Moths
Source: Sci Rep. 2019 Nov 11;9:16413. doi: 10.1038/s41598-019-52840-z (PMC6848174; doi:10.1038/s41598-019-52840-z)
Supplement: Supplementary file 1 — Supplementary information document [file 41598_2019_52840_MOESM1_ESM.pdf]

Expansion of LINEs and species-specific DNA repeats drives genome expansion in Asian Gypsy Moths.

Francois Olivier HEBERT[1*], Luca FRESCHI[1], Gwylim BLACKBURN[1], Catherine BÉLIVEAU[2], Ken DEWAR[3], Brian BOYLE[1], Dawn E. GUNDERSEN-RINDAL[4], Michael E. SPARKS[4], Michel CUSSON[1,2], Richard C. HAMELIN[1,5], Roger C. LEVESQUE[1]

1. Institut de Biologie Intégrative et des Systèmes (IBIS), Université Laval, Canada.
2. Laurentian Forestry Centre, Canadian Forest Service, Natural Resources Canada, Quebec City, Quebec, Canada.
3. Department of human genetics, McGill University, Montreal, Quebec, Canada.
4. United States Department of Agriculture - ARS Invasive Insect Biocontrol and Behavior Laboratory, Beltsville, Maryland, USA
5. Department of Forest and Conservation Sciences, Faculty of Forestry, University of British Columbia, Vancouver, British Columbia, Canada.

*Corresponding author: francois-olivier.gagnon-hebert.1@ulaval.ca

Supplementary Information – Table of Content

S1. SAMPLING, LIBRARY PREPARATION AND SEQUENCING **3**

S2. GENOME ASSEMBLY AND ANNOTATION PIPELINE **4**

In-house, multi-step pipeline **4**

BUSCO coverage assessment in AGM genomes **7**

S3. GENOME ASSEMBLY POST-PROCESSING & PAIRWISE COMPARISONS OF AGM GENOMES **7**

Clustering similar sequences into consensus gene models and genome polishing **7**

Identification of AGM orthologs **9**

S4. GENOME-WIDE CHARACTERIZATION OF DEVELOPMENTAL GENES **10**

S5. FLIGHTIN COMPARATIVE SEQUENCE ANALYSIS AND PCR VALIDATION **11**

SUPPLEMENTARY FIGURES **16**

Figure S1. Distribution of 31-mers in the AGM genomes. **16**

Figure S2. Read length distributions of the AGM subspecies sequenced in this study. **17**

Figure S3. Kyoto Encyclopedia of Genes and Genomes (KEGG) analysis on AGM gene models. **18**

Figure S4. Genome completeness assessed by the presence of BSCO groups in the genomes of four Lepidoptera species. **19**

Figure S5. Genome-wide diversity of homeodomain peptides in the Asian gypsy moth. **20**

Figure S6. *Flightin* (*fln*) gene sequence variants initially identified as potentially involved in gypsy moth flight capability. **21**

Figure S7. *Flightin* (*fln*) gene sequence variants confirmed as sequencing errors. **22**

REFERENCES **23**

S1. SAMPLING, LIBRARY PREPARATION AND SEQUENCING

Live specimens of *Lymantria dispar japonica* were collected in the in the Northern Iwate District of Japan in October of 2005, to be reared in the laboratory of Dr. Hannah Nadel from USDA-APHIS (Buzzard's Bay, MA). Live specimens of *Lymantria dispar asiatica* were collected in October of 2007 in Tianjin (China). Specimens were brought back in the same controlled laboratory conditions as *L. dispar japonica*, in the USDA-APHIS rearing facility (Buzzard's Bay, MA). Insects used in this study were provided by Dr. Nadel's laboratory as lab-raised adult moths (10th generation fed on artificial diet) frozen alive and kept at -80°C until DNA extraction. The reason we only sequenced males is because males are the homogametic sex (ZZ) in this species and the female W chromosome (ZW) is very difficult to sequence, so we wanted to maximize our chances of constructing a first genome reference as complete as possible. Sequencing insect genomes can be challenging due to their intrinsically high level of polymorphism, low DNA extraction yields, and difficulty to produce homozygous lineages [74]. Taking into account the amount of raw gDNA required for PacBio sequencing, genome sizes and complexities, desired coverage, sequencing costs, and the relatively low yields of DNA extraction methods obtained on adults, we pooled the gDNA of two random males per subspecies. Considering that we did not have access to AGM haploid lineages, this approach allowed us to obtain enough gDNA to generate adequate amounts of raw PacBio sequences, and at the same time, to keep the lowest possible number of diploid individuals per genome assembly (maximizing the quality of each genome assembly). The head, thorax and legs of two diploid *L. dispar asiatica* adult males and two diploid *L. dispar japonica* adult males were used respectively to perform total gDNA extraction, using Qiagen Blood & Cell Culture DNA kit (#13343, Germantown, MA, USA).

Raw gDNA extracted from these four adult insects (two pooled individuals per species) was used to prepare Pacific Biosciences (PacBio) sequencing libraries according to the manufacturer's protocol. A total of 70 and 45 size-selected SMRTcell libraries were prepared for *L. dispar asiatica* and *L. dispar japonica* respectively. Raw sequencing libraries for the two species were deposited into the NCBI Sequence Read Archive (SRA) with accession numbers SAMN09601828 and SAMN09601829, for *L. dispar asiatica* and *L. dispar japonica* respectively, and associated with BioProjects PRJNA479680 and PRJNA479831.

S2. GENOME ASSEMBLY AND ANNOTATION PIPELINE

In-house, multi-step pipeline

Raw sequencing reads for each *L. dispar* species were assembled into longer genomic sequences, investigated for repeated and low complexity elements, protein-coding sequences, gene products, and Gene Ontology (GO) terms in a multistep custom made pipeline (available at http://github.com/fohebert/GenomeAnnotation). The gypsy moth genome annotation pipeline used in this study ultimately returned gene structures with their corresponding functional annotation (protein-coding genes, KEGG pathways, GO terms), ready for downstream analyses or genome sequencing improvement (e.g. scaffolding with additional sequencing data, analysis of nucleotide polymorphisms, preliminary genome wide association studies).

The first step involved the assembly of raw sequencing reads into longer contig sequences. Two separate genome assemblies were performed in this study, i.e. one for each *Lymantria dispar* subspecies. For each of the two assemblies, raw PacBio sequencing reads from all libraries were assembled using Canu v.1.5 (genomeSize = 1.5g, maxMemory = 800, maxThreads = 60, all other parameters kept to default mode [55]). Three different stages in a Canu run, each implementing specific and complementary algorithms, allowed for the correction, trimming and assembly of raw reads. After each of the two gypsy moth genome assemblies, a quantitative measure of genome assembly completeness was computed using BUSCO v.3.0 with the Arthropoda gene set (available at https://busco.ezlab.org/).

The second step involved the creation of a *Lymantria*-specific repetitive DNA database using RepeatModeler v.1.0.8[56] ('-engine ncbi', all other parameters kept to default mode). RepeatModeler implements two complementary computational methods allowing *de novo* identification and modeling of interspersed repeat elements from genomics sequence data. The program analyzes a given genomic database in search of repeated elements and ultimately builds, refines and classifies consensus models of interspersed repeats, placed in a species-specific repeat database. This species-specific repeat database was then concatenated with the curated Repbase library of repeats[57] and used by the program RepeatMasker v.4.0.6[58] to screen the two *Lymantria dispar* genomes in search of interspersed repeats and low complexity DNA sequences. Genomic regions tagged by the program as repeated elements or low complexity sequences were masked (i.e. each nucleotide replaced by the letter 'N') and the masked version of each genome was subsequently used for the rest of the pipeline.

The third step involved the identification of putative protein-coding sequences in the genome through pairwise sequence comparison, using two complementary approaches implemented in the programs Exonerate v.2.4.0[59] and SciPio v.1.4.1[60]. Exonerate offers an automatic implementation of sequence alignment algorithms, allowing the identification of genomic regions exhibiting high sequence similarities with known and curated protein-coding sequences from the manually annotated and reviewed UniProtKB/Swiss-Prot database[75]. SciPio is a computational tool that performs sequence alignments based on the program BLAT[76] to identify precise gene structures in a given genome. It allows the identification of exon-intron borders and splice sites based on protein-coding sequence alignments onto the reference genome. Since Exonerate and SciPio implement different algorithms, they were used in parallel to maximize the chances of identifying protein-coding sequences in the two *Lymantria dispar* genomes.

The fourth step involved the alignment of mRNA transcripts from the closely related subspecies *L. dispar dispar*[10] to each of the two genomes using PASA v.2.1.0[61]. PASA (Program to Assemble Spliced Alignments) is a genome annotation tool that aligns transcript sequences to the genome using BLAT and then assembles clusters of overlapping transcript alignments into gene structures. This step exploits valuable empirical transcript data to locate expressed genes in the genome and delineate their precise structure. This step is similar to the alignment of protein sequences from public databases to the genome, only this time the results should be considered as more reliable, because the mRNA query sequences used by the program are specific to the genomes being annotated here and those sequences were empirically validated as expressed genes.

The fifth step involved the use of a self-training algorithm implemented in the program GeneMark-ES v.1.0[62], which performs *ab initio* gene identification on a genome-wide scale. GeneMark-ES exploits an unsupervised training algorithm that allows the identification of protein-coding genes in a eukaryotic genome, based solely on genomic sequences, with no *a priori* knowledge on gene structure or coding regions, and does not require curated training sets. This step represents an additional source of evidence that needs to be considered along with transcript expression and sequence homology when building coherent and extensive gene annotations.

The sixth step involved the use of EVidenceModeler (EVM) v.1.1.1[63] to compute weighted consensus gene structure annotations based on the evidence gathered through steps three to five (i.e. Exonerate, SciPio, PASA, and GeneMark-ES). EVM allowed the creation of a comprehensive list of *Lymantria dispar spp.* gene structures based on multiple sources of evidence (see https://github.com/fohebert/GenomeAnnotation for details on the exact parameters used in this study). The final output from EVM was then used to generate a set of "high-confidence gene predictions", defined as gene structures supported by all sources of evidence processed by EVM (*ab initio* predictions, species-specific transcript alignments, and protein alignments).

The seventh step involved the use of AUGUSTUS v.3.2.2[64], a program that implements a Generalized Hidden Markov Model (GHMM) identifying protein-coding regions in a given genome by considering both extrinsic and intrinsic information into account. AUGUSTUS predicts gene structures based on *ab initio* algorithms that are adjusted and fine-tuned by incorporating various extrinsic "hints" that reflect the specific nature of a given genome (e.g. mRNA and protein alignments to the genome). In order for the program to be the most accurate possible in identifying gene structures, it is best if its algorithm is "pre-trained" specifically for the species being investigated (in this case *Lymantria dispar*). Gene prediction accuracy highly depends on the quality of the gene set used to perform this training[64]. Here, the gene set that was used to train AUGUSTUS corresponds to the "high-confidence gene predictions" previously selected according to EVM results and supported by four different, but complementary approaches (Exonerate, SciPio, GeneMark-ES and PASA). Once the algorithm was trained and optimized specifically for *L. dispar*, the program was ran a second time on each genome, using evidence from protein and transcript alignments as hints for AUGUSTUS. Protein-coding sequences identified by AUGUSTUS in each genome were extracted from their respective Genome Feature Format (GFF) file, along with their corresponding amino acid sequence, and used in the next and final step of the pipeline.

In the eighth and final step, gene structures obtained with AUGUSTUS were further annotated with putative gene products and GO terms using gapped BLASTp alignments to the UniProtKB/Swiss-Prot. All amino acid sequences obtained with the final AUGUSTUS run were aligned to the Swiss-Prot database (e-value < $1x10^{-30}$), and the best blast result for each query sequence (i.e. lowest e-value and highest bit score), along with its corresponding GO terms, was

kept has putative gene product. Sequences with no hit on Swiss-Prot were ultimately aligned to NCBI's non-redundant database (e-value < $1x10^{-30}$) and one best blast result per query sequence was kept as putative gene product. Putative gene products and GO terms were then propagated to the gene structures identified in each genome and combined in a final Generic Feature Format (GFF3) annotation file.

BUSCO coverage assessment in AGM genomes

To assess genome assembly and annotation completeness, we examined orthologous groups with single-copy orthologs among arthropods in the AGM genomes, using BUSCO. We used BUSCO v.3.0 in house with default parameters and the arthropoda gene set (odb9, created on 2017-02-07) available on the BUSCO website (https://busco.ezlab.org/). Results showed near complete *L. dispar* spp. genomes, with in total 96.5% and 98.2% arthropod BUSCO groups retrieved for *L. dispar asiatica* (complete single [S] = 79.5%, complete duplicated [D] = 14.1%, fragmented [F] = 2.9%, missing [M] = 3.5%) and *L. dispar japonica* (S = 73%, D = 23.5%, F = 1.7%, M = 1.8%) respectively. BUSCO scores for AGM were similar to the results that we obtained with other Lepidopteran species, namely the silk moth (95.5% complete BUSCO groups retrieved), the wood-white butterflies (98.7% complete BUSCO groups retrieved), and the recently sequenced *L.d. dispar* (EGM) genome[12] (Supplementary Figure S4). This result suggests that the genome sequences obtained in this study are of sufficiently high quality to be considered as a valuable and near-complete resource for the scientific community. The fraction of duplicated genes however seems slightly inflated as compared to closely related butterfly species[77,78], which reinforces the possibility that some of the sequences were artificially duplicated during the assembly process due to high heterozygosity levels and/or high repeat content. Ultimately, this analysis suggests that the genome assembly obtained for *L. dispar japonica* is slightly more duplicated, but more complete and less fragmented than the one obtained for *L. dispar asiatica*.

S3. GENOME ASSEMBLY POST-PROCESSING & PAIRWISE COMPARISONS OF AGM GENOMES

Clustering similar sequences into consensus gene models and genome polishing

We decided to collapse similar gene sequences into a single reference "consensus" sequence to correct for artificially duplicated gene models. This strategy is based upon the high content of

repeated sequences observed in AGM genomes and its potential impact on the overestimation of true gene numbers. The amount of DNA required for PACBIO WGS also required an assembly based upon sequence data from multiple individuals of a highly polymorphic organism[74]. To address this, we clustered amino acid sequences based on sequence similarity using a greedy incremental clustering algorithm implemented in the program CD-HIT[65], with a 95% sequence identity cutoff, a bandwith alignment of 20 and a 0.5 cutoff for; minimal alignment coverage for the longer sequence (-aL option), minimal alignment coverage for the shorter sequence (-aS option), and minimal length similarities (-s option). All other parameters were kept to default. For each cluster generated with CD-HIT, only the consensus sequence (i.e. longest representative sequence of the cluster) was kept in the final genome assembly and annotation files. This filtering step resulted in decreased overall numbers of contigs (*L.d. asiatica* [Lda], from 9,116 to 8,189 contigs; *L.d. japonica* [Ldj], from 12,764 to 11,303 contigs), increased N50s (Lda, from 201.7 Kb to 211.8 Kb; Ldj, from 128 Kb to 137 Kb), N90s (Lda, from 66.5 Kb to 72.6 Kb; Ldj, from 76.4 Kb to 82 Kb), reduced gene sets (Lda, from 22,300 to 19,588 genes; Ldj, from 27,489 to 23,292 genes), while slightly reducing genome size (Lda, from 961 Mb to 920 Mb; Ldj, 1.06 Gb to 999 Mb). For the terminal post-processing step, we applied the arrow algorithm released by PacBio as part of the GenomicConsensus utility package (https://github.com/PacificBiosciences/GenomicConsensus). Specifically, we used the wrapper script 'pbalign' (algorithm options: --bestn 10 --minMatch 12 --maxMatch 30 --minSubreadLength 100 --minAlnLength 100 --minPctSimilarity 80 --minPctAccuracy 80 --hitPolicy randombest --randomSeed 1) to align raw PacBio reads against the curated set of contigs generated for each AGM subspecies. The arrow algorithm was applied to the sorted and indexed BAM files (one sorted BAM file for each AGM subspecies), resulting in the correction of almost 0.1% of all the nucleotides contained in each genome. All of the genes that were kept after this analysis were considered as final and official gene models.

These final AGM genomes were ultimately aligned against one another using MUMmer's nucmer alignment algorithm v.3.23, a program that lists the distance between insertions and deletions that produce the maximal scoring alignments between the two sets of sequences being compared[79]. This allowed us to evaluate their relative similarity, possible structural rearrangements and contiguity (parameters: minimum length of a cluster of matches = 65 bp, maximum gap between two adjacent matches = 90, minimum length of a single match = 20, total

length of unique sequences an alignment must have on the query side to be retained = 10,000). Whole genome alignments were ultimately visualized using the program Dot, an open source dot plot viewer (https://github.com/dnanexus/dot). Final AGM genome versions obtained after the initial *de novo* assembly, annotation, and post-processing refinement were labeled as v.0.3.

As part of the downstream post-processing steps, a k-mer analysis was performed to explore potential biases in the assemblies that could be related to the fact that we assembled multiple individuals, i.e. mainly the creation of allelic contigs resulting in an artificially inflated number of contigs and high heterozygosity. We fragmented both AGM genomes into 31-mers using Jellyfish v.2.2.3[80] and plotted the distribution of the number of copies of each 31-mer in each genome (Supplementary Figure S1). Results indicated that, in both genomes, 96% of the 31-mers that we generated could be found in one or two copies. More specifically, we obtained seven percent of 2-copy 31-mers in *L. dispar asiatica* and 10% in *L. dispar japonica*, while the rest of the 31-mers (< 2% in both genomes) were present in more than 3 copies. In both genomes, we found a low number of 31-mers that were present in >10,000 copies (< 0.01 %), which is suggestive of highly repeated sequences, as confirmed by our RepeatMasker analysis. The same k-mer analysis was also performed on the previous version of the genomes, i.e. before running the CD-HIT filtering step (Supplementary Figure S1). This yielded similar k-mer distributions, but with longer tails on the right side, indicating that the CD-HIT filtering step was efficient at discarding highly repeated sequences in the genomes (k-mers with > 1,000 copies). This result shows that eliminating highly similar gene copies using CD-HIT in a newly assembled genome allows for conservative filtering of potential false duplications, without loosing too much information. This suggests that the two AGM genomes generated here can be considered as good representative haploid genomes at the species level.

<u>Identification of AGM orthologs</u>

We added another layer of information on top of the gene models by characterizing orthologous genes between the two AGM genomes and other related taxa using two independent approaches. First, we took advantage of NCBI's BLASTp to conduct a reciprocal best hit (RBH) analysis on the final genomes. We used pre-defined and empirically tested BLASTp options to optimize the detection of RBH, including a maximum e-value threshold of $1x10^{-6}$, a full Smith-Waterman alignment[81] (option '-use_sw_tback') and a coverage of at least 50% of the length of any

sequence in the alignment[82,83]. Orthologs added an additional confidence level to the gene models obtained with our pipeline. For example, if a predicted gene returned poor sequence homology with an unknown or uncharacterized protein in NCBI's non-redundant database, but was tagged as orthologous to a sequence from a closely related species, it is more likely that this gene is truly present in the genome. Second, we extended the scope of the orthology analysis by adding 12 extra lepidopteran species in a comprehensive orthogroup finding algorithm implemented in the program orthoFinder[66]. We used the final gene models obtained after the CD-HIT analysis and compared their amino acid sequences to the predicted proteomes of 12 butterfly and moth species, including *Plutella xylostella*, *Bombyx mori*, *Manduca sexta*, *Lerrema accius*, *Calycopis cecrops*, *Danaux plexippus*, *Heliconius melpomene melpomene*, *Heliconius erato lativitta*, *Melitaea cinxia*, *Papilio polytes*, *Papilio xuthus*, *Papilio glaucus* (amino acid sequences were downloaded from http://ensembl.lepbase.org/index.html). The 14 predicted proteomes were first used by orthoFinder to conduct all-vs-all BLASTp searches. BLASTp scores for each all-vs-all pairwise comparison were transformed into normalized BLAST bit scores, creating an unbiased comparison score that is independent of sequence length and that also normalizes for phylogenetic distance between species[66]. Length and phylogenetic distance normalized scores were used to cluster similar sequences based on reciprocal best normalized hits (RBNH). An orthogroup graph was built based on the RBNH scores and this graph was ultimately used by the Markov Cluster Algorithm (MCL)[84] to create the final orthogroups and rooted species tree. We used the pairwise comparison matrixes generated by the orthoFinder program for many-to-one orthologous relationships to construct a heatmap with the R package 'corrplot'[85].

S4. GENOME-WIDE CHARACTERIZATION OF DEVELOPMENTAL GENES

ANTP (derived from *Antennapedia* gene originally found in *Drosophila melanogaster*) is the most abundant homeobox gene class in AGM genomes. In total, 33 *L.d. asiatica* and 42 *L.d. japonica* homeobox genes belonging to the ANTP class were retrieved, each spanning 24 unique families (Figure S5, Tables S8 & S9). These ANTP gene counts are within the range observed in other insect species (*Drosophila melanogaster* [47 genes]; the red flour beetle; *Tribolium castaneum* [45 genes]; and the honeybee; *Apis mellifera* [39 genes]; HomeoDB[2]; http://homeodb.cbi.pku.edu.cn/) but considerably less abundant than those observed in vertebrates (human, *Homo sapiens* [100 genes]; mouse, *Mus musculus* [100]; chicken, *Gallus*

gallus [68 genes]; zebrafish, *Danio rerio* [129 genes]; and frog, *Xenopus tropicalis* [112 genes]). Previous work on ANTP homeobox genes in *Bombyx mori* identified 55 genes belonging to 32 families, with a unique species-specific expansion of 12 tandem duplicated *Bmshx* genes that are part of the hox cluster[86]. This organizational pattern of hox genes in *B. mori* represents an exception, as duplications in hox genes have occurred only rarely during lepidopteran evolution[87]. The duplicated *Bmshx* genes were not detected in either of the two AGM genome assemblies.

A striking feature of developmental genes in Lepidoptera is the presence of four *Shx* genes that originated from tandem duplications of the *zen* gene early in ditrysian evolution. These duplicated genes have been retained in all descendent Lepidoptera except for *B. mori*[88]. Our results suggest that these ancestral tandem duplications of *zen* are also present in AGM, as we identified four and five highly similar zen-like genes in the genomes of *L.d.* asiatica and *L.d. japonica*, respectively (Supplementary Tables S8 & S9). Since these genes are highly conserved among diverse lepidopteran taxa, the presence of only four zen-like genes in *L.d. asiatica* could mean that we simply missed one of the copies during the assembly. The presence of multiple zen-like genes in AGM emphasizes the importance of developmental gene conservation throughout evolution. It is important to note that the inherent short length of homeobox domains and their high sequence similarity with one another in the context of highly repeated genomes might have impaired our ability to retrieve certain classes of homeotic genes as monophyletic groups. Additional sequencing efforts will be invested in upcoming genome versions in order to better characterize this set of genes. This further highlights the complexity of classifying short homeotic genes into circumscribed categories that relate to their structure and biological function based on sequence similarity alone.

S5. FLIGHTIN COMPARATIVE SEQUENCE ANALYSIS AND PCR VALIDATION

Despite significant progress in understanding the underpinnings of flight, it is still unclear exactly how, from a molecular perspective, to predict female flight ability. Identifying a specific genomic signature related to flight capability would be of great interest in the management of AGM outbreaks in North America, and more generally in the understanding of molecular determination of flight in insects. Previous studies showed that flightless EGM females from North America tend to exhibit decreased muscle strength as compared to flight-capable Russian AGM females,

which is due in part to a different muscle fiber organization and composition[8]. In *Drosophila melanogaster*, mutations in the coding sequence of the *flightin* (*fln*) gene, which encodes a myosin rod binding protein[89], can affect the proper assembly and functioning of indirect flight muscles[90-92]. In that species, a null mutation in *fln* was shown to disrupt thick filament assembly, resulting in compromised muscle integrity and ultimately, in the loss of flight capability[93,94].

Despite the likely polygenic nature of flight in gypsy moth[95], the *D. melanogaster* example suggests that a genetic defect in a single gene may result in substantial or total loss of flight capability. We explored this possibility in AGM. We firstly searched the genome annotation files generated through our pipeline for *L. dispar asiatica* and *L. dispar japonica* to see if *fln* was present in any of the two genomes. We found one gene sequence in each genome that was annotated as *fln*. The level of synteny between Z chromosomes in butterflies and moths is high, which facilitates the assignment of gene sequences to sexual chromosomes or autosome through orthology relationships[96,97]. Using our AGM-specific *fln* sequences, we conducted a BLASTx search against the most recent version of the *B. mori* genome (http://silkbase.ab.a.u-tokyo.ac.jp/cgi-bin/download.cgi). BLAST results returned positive hits on the Z chromosome of *B. mori* (*L.d. asiatica*: identities = 69%, bit score = 127; *L.d. japonica*: identities = 67%, bit score = 127), which suggests that *fln* could also be located on AGM sex chromosomes.

Work conducted on *Bombyx mori* and *Manduca sexta*, the two most closely related moth species to AGM[98], revealed the existence of extensive gene dosage compensation between males and females. Data on dosage compensation in these two moth species also confirmed that major sexually dimorphic phenotypes were associated with sex-specific gene expression profiles[97]. Based on these pieces of information, we postulated that a recessive, Z-linked loss-of-function mutation in the *fln* gene could be involved in the expression of a flightless phenotype in female AGM. A dosage compensation effect enabling male flight in the presence of two mutated flight genes, or the evolution of another mechanism (e.g. additional flight-related genes, or the presence of modifier genes that could rescue male flight despite a compromised *fln* coding sequence) could explain the dimorphic nature of this trait.

To test this possibility within the *fln* gene itself, we extracted the corresponding *fln* nucleotide coding sequences and amino acid sequences and used them to find the EGM-specific *fln* gene sequence in the transcriptome and predicted proteome of *Lymantria dispar dispar* (NCBI BioProject accession #PRJNA76163), using BLASTn and BLASTp v.2.6.0[69]. We aligned the *fln* nucleotide sequences for the three *Lymantria dispar* species using CLUSTAL-OMEGA v.1.2.4[72] with default parameters in search of Single Nucleotide Polymorphisms (SNPs) and/or small insertion-deletions (INDELs) in the gene sequence that could explain differences in flight capabilities between AGM and EGM. This preliminary sequence comparison analysis between AGM and EGM revealed two potential INDELs (positions 118 in exon 1 and position 607 in exon 2) that could be responsible for a putative loss of function frame-shift in the Open Reading Frame (ORF) (Supplementary Figure S5). This frame-shift observed in the *fln* coding sequence found in a transcriptome of *Lymantria dispar dispar* resulted in two possible mRNA translations, both of which had a truncated amino acid sequence (82 and 86 amino acids respectively), as compared to the translated coding sequences obtained in our AGM genome assemblies (155 amino acids in both AGM ssp.) (Supplementary Figure S6). The presence of a truncated and potentially non-functional *fln* protein in gypsy moth females could explain the absence of flight in this *Lymantria dispar* subspecies. To validate genetic variations detected in the alignment of AGM (flight-capable females) and EGM (flightless females) *fln* gene sequences, we conducted a Polymerase Chain Reaction (PCR) amplification in all three *Lymantria dispar* species (*L. dispar dispar*, *L. dispar asiatica* and *L. dispar japonica*), using gDNA samples extracted from specimens collected at different geographic locations (Supplementary Table S1). Fresh insect tissues frozen at -80°C were obtained from Dr. Melody Keena at USDA Forest Service (Northern Research Station, Hamden, Connecticut, USA), including males (*L. dispar asitica*: n = 3, *L. dispar dispar*: n = 5) and females (*L. dispar asitica*: n = 5, *L. dispar dispar*: n = 5, *L. dispar japonica:* n = 5). Genomic DNA extractions were performed with Qiagen DNeasy Blood & Tissue kit spin-column protocol for animal tissue, following the default manufacturer's procedure with an overnight incubation at 56°C. *Flightin* sequences were amplified using custom primers targeting 100 bp upstream and 51 bp downstream of the gene, for a total amplicon length of 994 bp:

AGM-*fln*-F = 5' AACCCAGTTAGGTCTGTGAAGTGAC 3'

AGM-*fln*-R = 5' TTGTATCGTTCAGGTAGCTGTGGCAG 3'

PCR amplifications were prepared in a 25µL reaction volume containing 10 ng of template gDNA, 1U of Taq polymerase (Kapa high fidelity, KapaBiosystems), and a final concentration of 200µM dNTPs (KapaBiosystems), 0.5µM of forward and reverse AGM-*fln* primers, and 1X PCR buffer (KapaBiosystems). PCR program consisted of an initial denaturation at 95°C for 3 minutes, followed by 35 cycles of 15s denaturation at 95°C, 15s annealing at 60°C and 15s extension at 72°C, and a final extension time of 60s at 72°C after the 35 cycles. PCR products were visualized by electrophoresis on a 1.25% agarose gel. All PCR products were sent to Plateforme d'analyses génomiques (Institut de Biologie Intégrative et des Systèmes, Université Laval, Québec) for Sanger sequencing. Resulting *flightin* nucleotide sequences were finally aligned using CLUSTA-OMEGA v.1.2.4[72] and visualized with CLC Sequence Viewer v.7.8.1 (QIAGEN Bioinformatics).

Final nucleotide alignments of population-specific *fln* amplicons suggested that variable flight capabilities in *Lymantria dispar ssp.* were not related to indirect flight muscle assembly. Our PCR and Sanger sequencing validations of two putative INDELs in multiple populations of *Lymantria dispar dispar*, *Lymantria dispar asiatica* and *Lymantria dispar japonica* (Table S1) confirmed that the two INDELs identified in the *fln* mRNA sequence from the EGM transcriptome are most likely sequencing errors (Supplementary Figure S7). A multiple sequence alignment of the *fln* PCR amplicons revealed the presence of some polymorphism within and among gypsy moth populations, mainly synonymous substitutions, but no INDELs were detected in any of the exons and, in particular, none were found in exons 1 and 2 (Supplementary Figure S7). The absence of a structural variation in *fln* does not rule out the possibility that mutations in regulatory regions of *fln* (or affecting other functionally related genes) disrupt flight muscle function or other crucial traits involved in flight. AGM and EGM female flight phenotype data, combined with expression data or markers (e.g., SNPs, indels, copy number variants) representing developmental genes, will aid further investigation of this issue.

This PCR validation allowed us to test the quality of our genome assemblies and their quality as reference sequences. We showed that it is possible to mine the AGM genomes generated here in search of specific full-length gene targets, and use them as references for downstream functional

analyses. Considering that PacBio sequencing generates single pass error rates of ~10-15% with a final consensus accuracy > 99%[99,100], with careful experimental design, sufficient read depth and adequate analytical pipeline, it is possible to achieve good accuracy to generate high quality genomic references.

SUPPLEMENTARY FIGURES

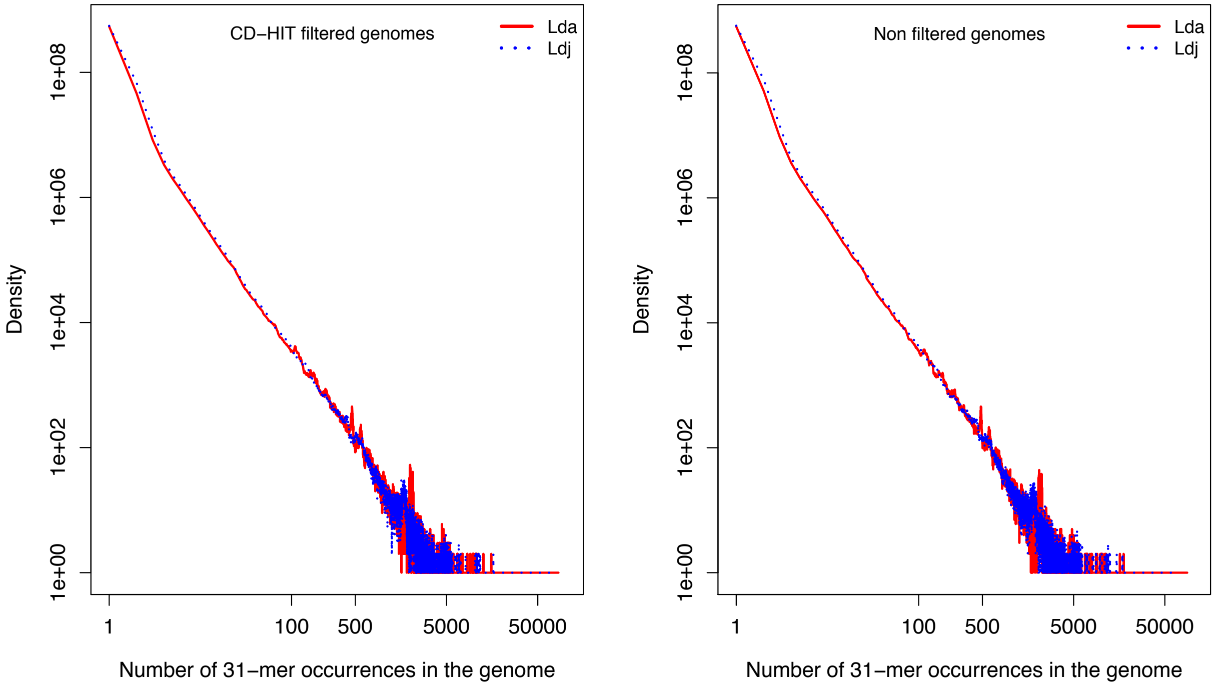

Figure S1 Distribution of 31-mers in AGM genomes. Right: distribution of 31-mers across unfiltered AGM genomes. Left: distribution of 31-mers across CD-HIT filtered AGM genomes. Number of single-copy 31-mers in *L.d. asiatica* = 5.42×10^8 (89% of all 31-mers). Number of single-copy 31-mers in *L.d. japonica* = 5.54×10^8 (85% of all 31-mers). Largest 31-mer occurrence across *L.d. asiatica*: CD-HIT filtered = 84,228, non-filtered = 87,936. Largest 31-mer occurrence across *L.d. japonica*: CD-HIT filtered = 66,636 non-filtered = 70,645. Solid red line = *Lymantria dispar asiatica*, dotted blue line = *Lymantria dispar japonica*.

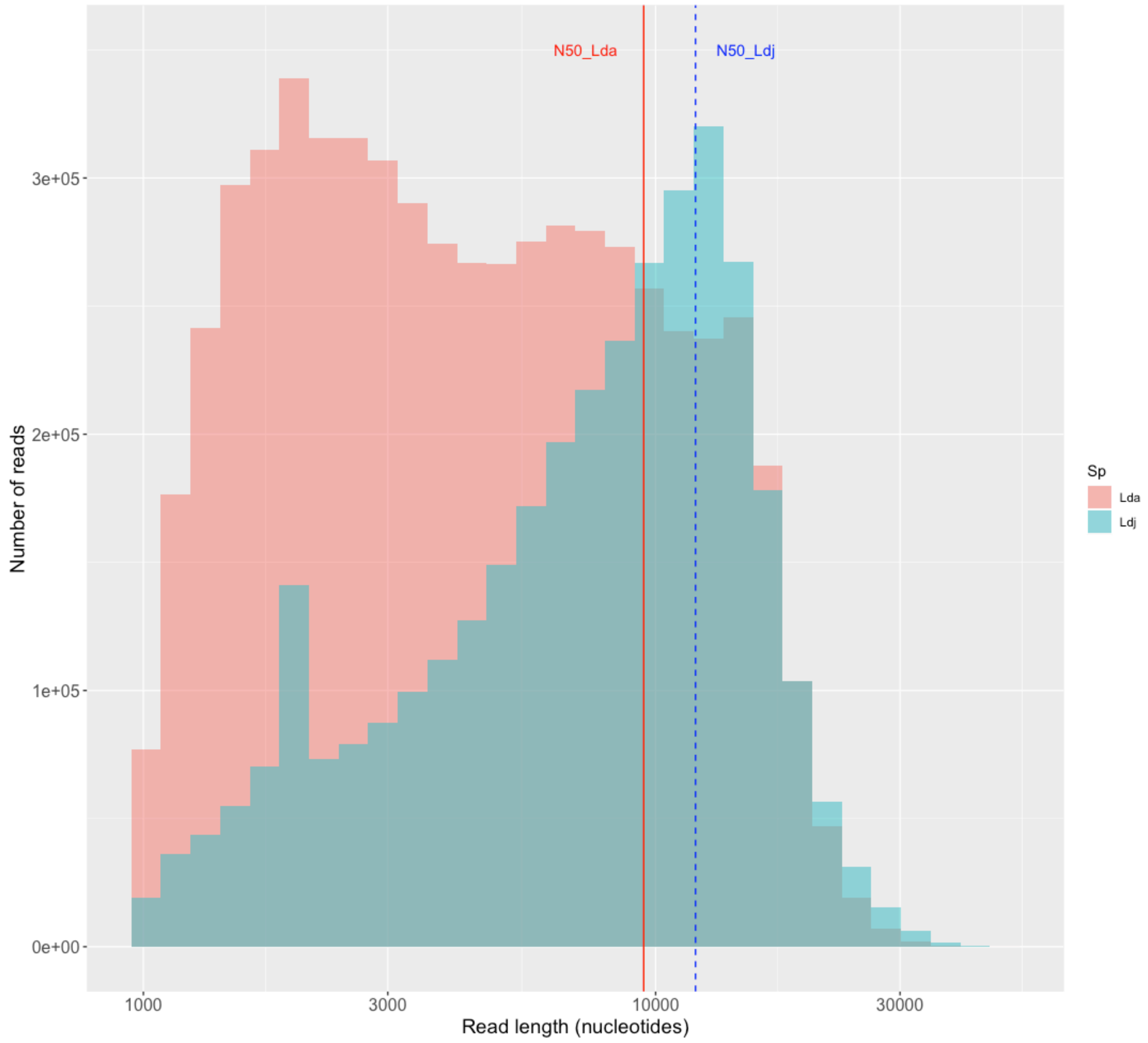

Figure S2 Read length distributions of the AGM subspecies sequenced in this study. Solid red vertical line = N50 of all read lengths for *L. dispar asiatica* (in this case, N50 = 9,478). Dashed blue line = N50 of all read lengths for *L. dispar japonica* (in this case, N50 = 11,963). Legend: Sp = species, Lda = *Lymantria dispar asiatica*, Ldj = *Lymantria dispar japonica*.

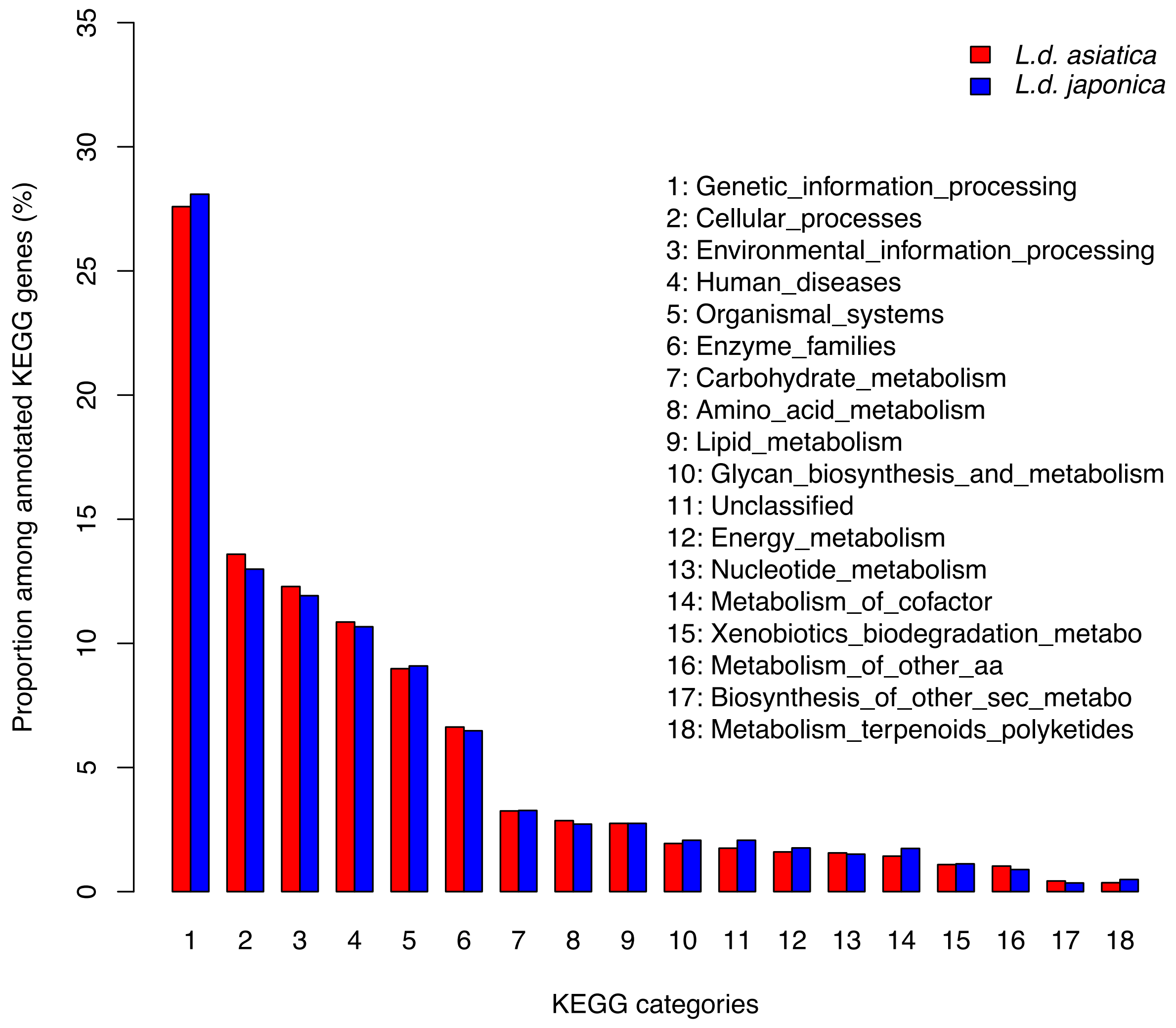

Figure S3 Kyoto Encyclopedia of Genes and Genomes (KEGG) analysis on AGM protein-coding gene models. Distribution of KEGG Orthology (KO) categories represented in the genome assemblies of *L. dispar asiatica* and *L. dispar japonica*. In total, 28% of all the protein-coding genes could be annotated with at least one KEGG number in *L. dispar asiatica*, while 32% of all the protein-coding genes from the genome of *L. dispar japonica* could be annotated with at least one KEGG number. Proportion among annotated KEGG genes (%) = number of genes annotated in a given KEGG pathway / all KEGG-annotated genes.

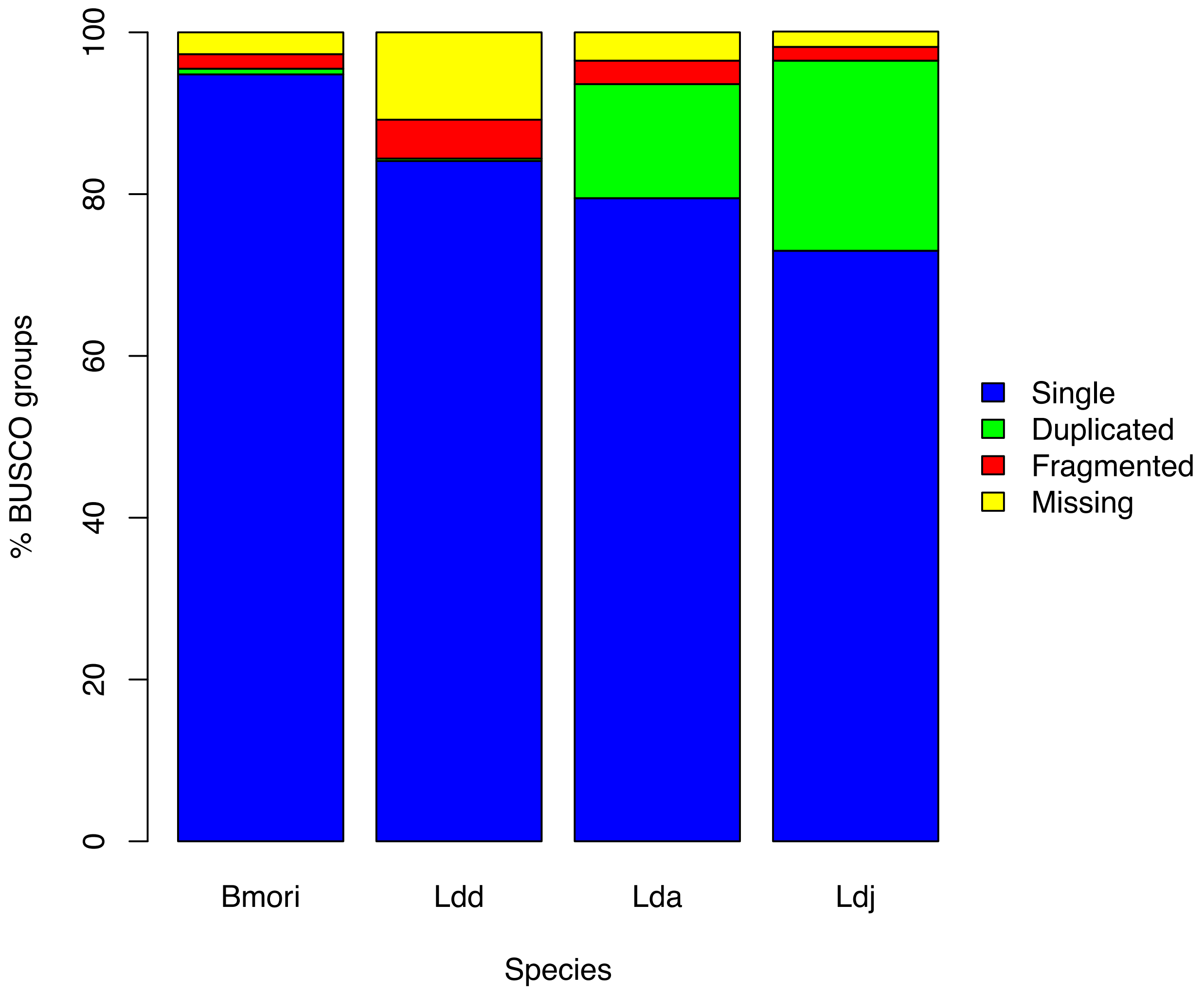

Figure S4 Genome completeness assessed by the presence of BUSCO groups in the genomes of four Lepidoptera species. Bmori = *Bombyx mori*, Ldd = *Lymantria dispar dispar*, Lda = *Lymantria dispar asiatica*, Ldj = *Lymantria dispar japonica*. Searches were conducted on the final genome versions of *L. dispar asiatica* and *L. dispar japonica*, while genome sequences used for *B. mori* and *L. dispar dispar* were downloaded from LepBase Ensembl genome database (http://ensembl.lepbase.org/) and NCBI, respectively. Scores for Bmori: complete single [S] = 94.8%, complete duplicated [D] = 0.7%, fragmented [F] = 1.8%, missing [M] = 2.7%. Scores for Ldd: complete single [S] = 84.1%, complete duplicated [D] = 0.3%, fragmented [F] = 4.8%, missing [M] = 10.8%. Scores for Lda: complete single [S] = 79.5%, complete duplicated [D] = 14.1%, fragmented [F] = 2.9%, missing [M] = 3.5%. Scores for Ldj: [S] = 73%, [D] = 23.5%, [F] = 1.7%, [M] = 1.8%.

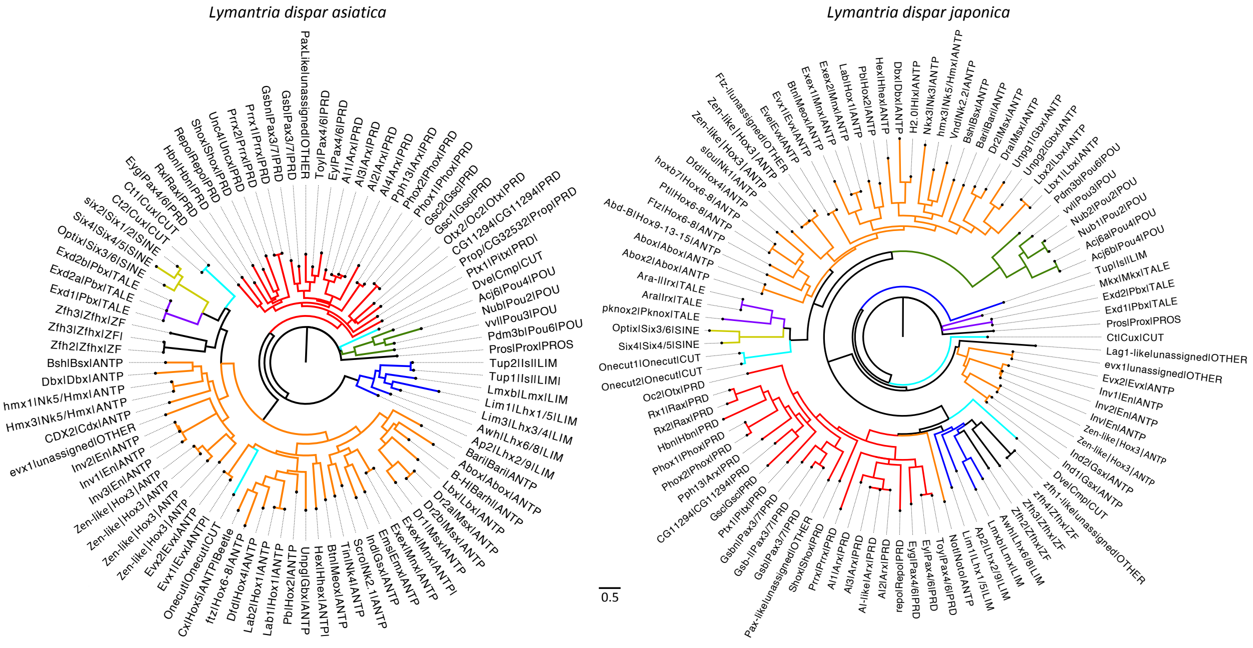

Figure S5 Genome-wide diversity of homeodomain peptides in the Asian gypsy moth. Phylogenetic analysis of all the genes containing at least one putative homeodomain peptide sequence in the genomes of *L.d. asiatica* (left) and *L.d. japonica* (right). Genes are labeled according to their respective homeodomain gene ID, family, and class, according to classification implemented in HomeoDB[2], a homeobox gene database. Colors in the phylogenetic networks indicate the class to which a gene belongs: ANTP (orange), CUT (turquoise), LIM (blue), PRD (red), POU (green), SINE (yellow), TALE (purple), ZF (black).

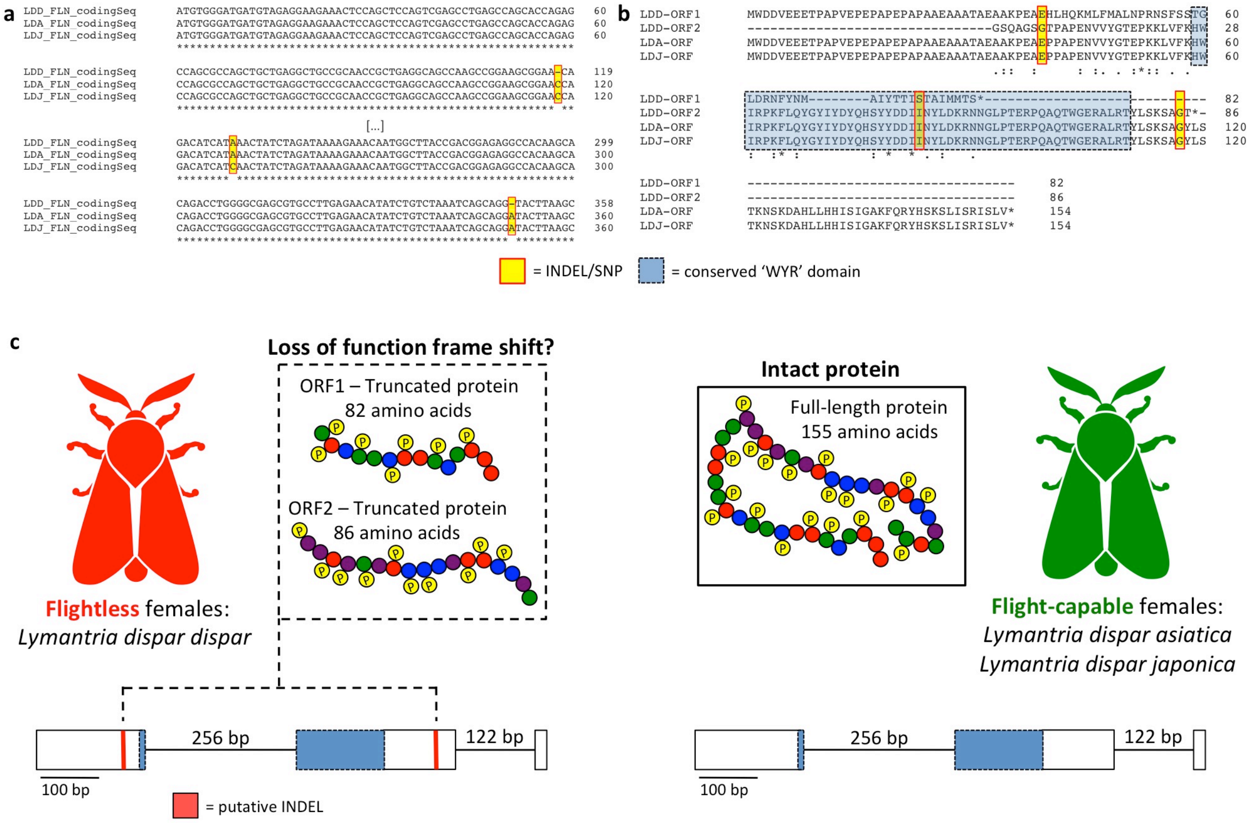

Figure S6 *Flightin* (*fln*) gene sequence variants initially identified as potentially involved in gypsy moth flight capability. **A)** Initial multiple alignment of the coding sequence of *fln* taken from a published transcriptome of *Lymantria dispar dispar* (LDD) and the genomes of *Lymantria dispar asiatica* (LDA) and *Lymantria dispar japonica* (LDJ) assembled and annotated in this study. This alignment revealed two putative insertion-deletions (INDELs) and one Single Nucleotide Polymorphism (SNP) between LDD (European Gypsy Moth, EGM) and LDA/LDJ (Asian Gypsy Moths, AGM). **B)** Protein alignment generated with the translated sequences shown in A. WYR domain = highly conserved domain widespread in Pancrustacean and spanning 52 amino acids rich in tyrosine (Y) bound by strict conservation of tryptophan (W) and arginine (R). **C)** The two INDELs identified in the EGM-specific mRNA sequence could potentially generate truncated and potentially non-functional proteins, producing flightless European gypsy moth females.

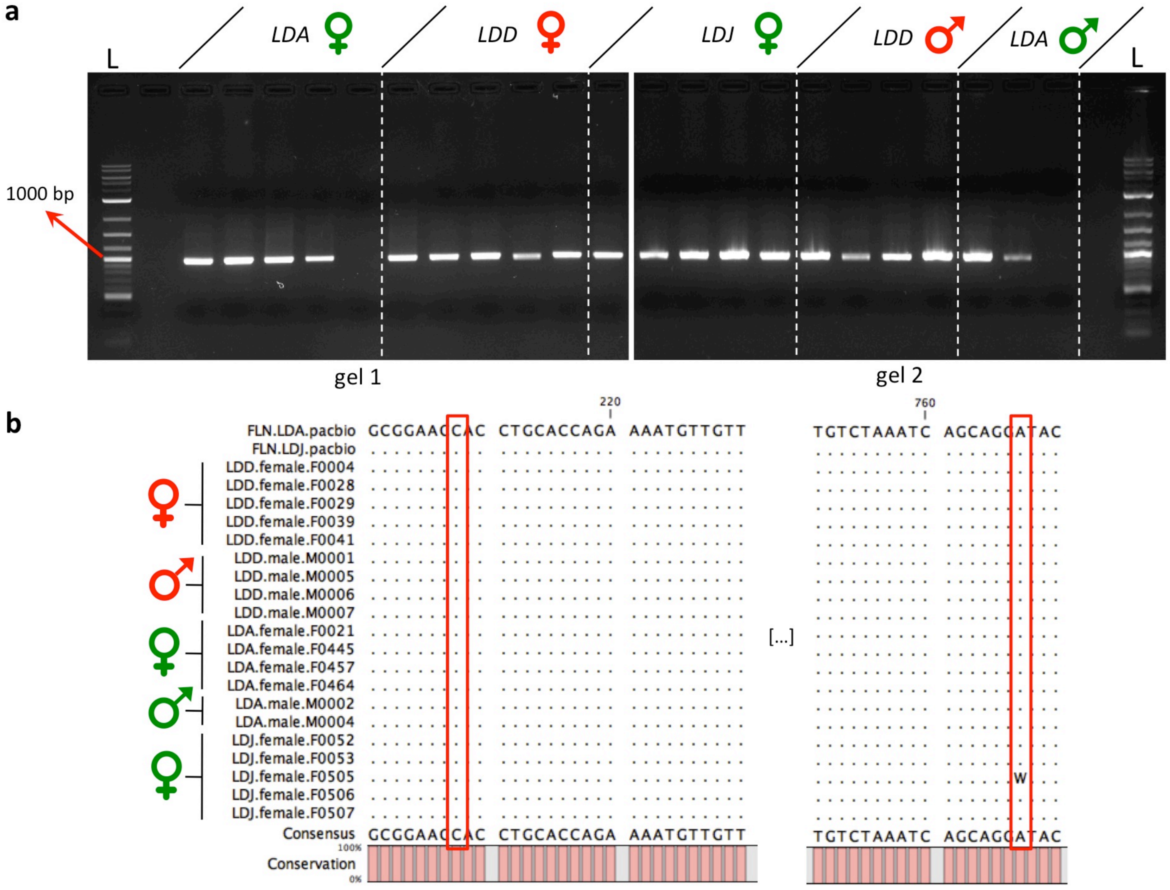

Figure S7 *Flightin* (*fln*) gene sequence variants confirmed as sequencing errors. A) Digital images of the gels electrophoresis used to visualize the results of a Polymerase Chain Reaction (PCR) conducted on gDNA of male and female gypsy moths from different sub-species used to amplify the *fln* gene. Gel 1 (left) and gel 2 (right) were prepared, ran a

nd visualized on the same day. The complete gene sequence totalizes 843 bp and the total length of the PCR product amplified with our custom-made primers is 994 bp. Only two samples did not amplify the gene, i.e. one *Lymantria dispar asiatica* female and one *Lymantria dispar asiatica* male. All other samples showing a single amplicon of the target size were sequenced with the Sanger method and analyzed to confirm the presence of two putative INDELs. B) Alignment of the sequenced PCR amplicons obtained in females and males gypsy moth from multiple populations confirmed that the putative INDELs were sequencing errors. LDA = *Lymantria dispar asiatica*, LDD = *Lymantria dispar dispar*, LDJ = *Lymantria dispar japonica*. Green = species belonging to the Asian Gypsy Moth species complex, red = European Gypsy Moth. L = Ladder.

REFERENCES

74. Richards, S. & Murali, S. C. Best practices in insect genome sequencing: what works and what doesn't. *Curr Opin Insect Sci* **7,** 1–7 (2015).
75. The UniProt Consortium. UniProt: the universal protein knowledgebase. *Nucleic Acids Res* **45,** D158–D169 (2017).
76. Kent, W. J. BLAT--the BLAST-like alignment tool. *Genome Res* **12,** 656–664 (2002).
77. Consortium, T. H. G. & Consortium, G. Butterfly genome reveals promiscuous exchange of mimicry adaptations among species. *Nature Publishing Group* **487,** 94–98 (2012).
78. Cong, Q. *et al.* Speciation in cloudless sulphurs gleaned from complete genomes. *Genome Biol. Evol.* **8,** 915–931 (2016).
79. Kurtz, S. *et al.* Versatile and open software for comparing large genomes. *Genome Biol* **5,** R12–9 (2004).
80. Marçais, G. & Kingsford, C. A fast, lock-free approach for efficient parallel counting of occurrences of k-mers. *Bioinformatics* **27,** 764–770 (2011).
81. Smith, T. F. & Waterman, M. S. Identification of common molecular subsequences. *J Mol Biol* **147,** 195–197 (1981).
82. Moreno-Hagelsieb, G. & Latimer, K. Choosing BLAST options for better detection of orthologs as reciprocal best hits. *Bioinformatics* **24,** 319–324 (2008).
83. Ward, N. & Moreno-Hagelsieb, G. Quickly finding orthologs as reciprocal best hits with BLAT, LAST, and UBLAST: how much do we miss? *Plos One* **9,** e101850–6 (2014).
84. Enright, A. J., Van Dongen, S. & Ouzounis, C. A. An efficient algorithm for large-scale detection of protein families. *Nucleic Acids Res* **30,** 1575–1584 (2002).
85. Zheng, T., Salganik, M. J. & Gelman, A. How many people do you know in prison? *Journal of the American Statistical Association* **101,** 409–423 (2006).
86. Chai, C.-L. *et al.* A genomewide survey of homeobox genes and identification of novel structure of the Hox cluster in the silkworm, *Bombyx mori*. *Insect Biochem. Mol. Biol.* **38,** 1111–1120 (2008).
87. de Rosa, R. *et al.* Hox genes in brachiopods and priapulids and protostome evolution. *Nature* **399,** 772–776 (1999).
88. Ferguson, L. *et al.* Ancient expansion of thehHox cluster in Lepidoptera generated four homeobox genes implicated in extra-embryonic tissue formation. *Plos Genet* **10,** e1004698–12 (2014).
89. Ayer, G. & Vigoreaux, J. O. Flightin is a myosin rod binding protein. *Cell Biochem. Biophys.* **38,** 41–54 (2003).
90. Vigoreaux, J. O., Hernandez, C., Moore, J., Ayer, G. & Maughan, D. A genetic deficiency that spans the flightin gene of Drosophila melanogaster affects the ultrastructure and function of the flight muscles. *J. Exp. Biol.* **201,** 2033–2044 (1998).
91. Reedy, M. C., Bullard, B. & Vigoreaux, J. O. Flightin is essential for thick filament assembly and sarcomere stability in Drosophila flight muscles. *J Cell Biol* **151,** 1483–1500 (2000).
92. Contompasis, J. L., Nyland, L. R., Maughan, D. W. & Vigoreaux, J. O. *Flightin* is necessary for length determination, structural integrity, and large bending stiffness of insect flight muscle thick filaments. *J Mol Biol* **395,** 340–348 (2010).
93. Henkin, J. A., Maughan, D. W. & Vigoreaux, J. O. Mutations that affect *flightin* expression in Drosophila alter the viscoelastic properties of flight muscle fibers.

American Journal of Physiology-Cell Physiology **286,** C65–C72 (2004).
94. Barton, B. *et al.* Flight muscle properties and aerodynamic performance of Drosophila expressing a *flightin* transgene. *J. Exp. Biol.* **208,** 549–560 (2005).
95. Keena, M. A., Grinberg, P. S. & Wallner, W. E. Inheritance of female flight in *Lymantria dispar* (Lepidoptera: Lymantriidae). *Environ. Entomol.* **36,** 484–494 (2007).
96. Harrison, P. W., Mank, J. E. & Wedell, N. Incomplete sex chromosome dosage compensation in the Indian meal moth, *Plodia interpunctella*, based on *de novo* transcriptome assembly. *Genome Biol. Evol.* **4,** 1118–1126 (2012).
97. Smith, G., Chen, Y.-R., Blissard, G. W. & Briscoe, A. D. Complete dosage compensation and sex-biased gene expression in the moth *Manduca sexta*. *Genome Biol. Evol.* **6,** 526–537 (2014).
98. Mitter, C., Davis, D. R. & Cummings, M. P. Phylogeny and evolution of Lepidoptera. *Annu. Rev. Entomol.* **62,** 265–283 (2017).
99. Chin, C.-S. *et al.* Nonhybrid, finished microbial genome assemblies from long-read SMRT sequencing data. *Nature Methods* **10,** 563–569 (2013).
100. Buermans, H. P. J. & Dunnen, den, J. T. Next generation sequencing technology: Advances and applications. *Biochim Biophys Acta* **1842,** 1932–1941 (2014).
